# Characterisation of an inflammation-related epigenetic score and its association with cognitive ability

**DOI:** 10.1186/s13148-020-00903-8

**Published:** 2020-07-27

**Authors:** Anna J. Stevenson, Daniel L. McCartney, Robert F. Hillary, Archie Campbell, Stewart W. Morris, Mairead L. Bermingham, Rosie M. Walker, Kathryn L. Evans, Thibaud S. Boutin, Caroline Hayward, Allan F. McRae, Barry W. McColl, Tara L. Spires-Jones, Andrew M. McIntosh, Ian J. Deary, Riccardo E. Marioni

**Affiliations:** 1 Centre for Genomic and Experimental Medicine, Institute of Genetics and Molecular Medicine, University of Edinburgh, Edinburgh, EH4 2XU UK; 2 UK Dementia Research Institute, Edinburgh Medical School, University of Edinburgh, Edinburgh, UK; 3 Centre for Discovery Brain Sciences, University of Edinburgh, Edinburgh, UK; 4 Medical Research Council Human Genetics Unit, Institute of Genetics and Molecular Medicine, Western General Hospital, University of Edinburgh, Edinburgh, EH4 2XU UK; 5 Institute for Molecular Bioscience, University of Queensland, Brisbane, QLD Australia; 6 Division of Psychiatry, Royal Edinburgh Hospital, University of Edinburgh, Edinburgh, EH10 5HF UK; 7 Lothian Birth Cohorts, University of Edinburgh, Edinburgh, EH8 9JZ UK; 8 Department of Psychology, University of Edinburgh, Edinburgh, EH8 9JZ UK

**Keywords:** Epigenetics, DNA methylation, C-reactive protein, Inflammation, Cognitive ability

## Abstract

**Background:**

Chronic systemic inflammation has been associated with incident dementia, but its association with age-related cognitive decline is less clear. The acute responses of many inflammatory biomarkers mean they may provide an unreliable picture of the chronicity of inflammation. Recently, a large-scale epigenome-wide association study identified DNA methylation correlates of C-reactive protein (CRP)—a widely used acute-phase inflammatory biomarker. DNA methylation is thought to be relatively stable in the short term, marking it as a potentially useful signature of exposure.

**Methods:**

We utilise a DNA methylation-based score for CRP and investigate its trajectories with age, and associations with cognitive ability in comparison with serum CRP and a genetic CRP score in a longitudinal study of older adults (*n* = 889) and a large, cross-sectional cohort (*n* = 7028).

**Results:**

We identified no homogeneous trajectories of serum CRP with age across the cohorts, whereas the epigenetic CRP score was consistently found to increase with age (standardised *β* = 0.07 and 0.01) and to do so more rapidly in males compared to females. Additionally, the epigenetic CRP score had higher test-retest reliability compared to serum CRP, indicating its enhanced temporal stability. Higher serum CRP was not found to be associated with poorer cognitive ability (standardised *β* = − 0.08 and − 0.05); however, a consistent negative association was identified between cognitive ability and the epigenetic CRP score in both cohorts (standardised *β* = − 0.15 and − 0.08).

**Conclusions:**

An epigenetic proxy of CRP may provide a more reliable signature of chronic inflammation, allowing for more accurate stratification of individuals, and thus clearer inference of associations with incident health outcomes.

## Background

Cognitive impairment in older age is associated with an increased risk of morbidity and mortality, and a lower quality of life [1–3]. Given the generally ageing population, and the significant personal and public health burden of age-related cognitive decline, insight into its determinants and the factors contributing to individual differences is critical. The strongest known risk factor for cognitive decline is older age, suggesting a unique facet of the ageing process is likely implicated.

Epidemiological studies have associated ageing with a progressive shift to a chronic inflammatory state. This low-grade, typically sub-acute, elevation of peripheral pro-inflammatory mediators in the absence of overt infection is strongly associated with the susceptibility to, and progression of, many age-associated diseases and is a key risk factor for mortality [4–6]. Accumulating evidence has also implicated chronic inflammation with incident dementia, but the association with pre-morbid cognitive function is less firmly defined and has generated considerable debate [7]. Evidence from large, prospective cohort and cross-sectional studies has been largely mixed, with positive, negative, and null associations identified between serum inflammatory biomarker levels and cognitive ability [8–12].

These conflicting findings may be attributed to methodological disparity between studies, including marked differences in both the ages of participants and type of cognitive assessment batteries used. However, a key issue in research utilising inflammatory biomarkers is their typically phasic responses [4]. C-reactive protein (CRP)—an acute-phase reactant of hepatic origin—is a widely used marker of inflammation. However, by definition, the plasma concentration of acute-phase proteins deviates by 25% or more in inflammatory disorders [13]. In particular, CRP is subject to swift and considerable shifts in response to injury or acute infection. Serum levels can rapidly increase up to 1000-fold from baseline, typically resolving to basal concentrations over a period of 7–12 days [14, 15]. This poses a potential issue when utilising CRP to investigate the association between chronic inflammation and health outcomes: the biological variability of the biomarker may not be stable enough to enable reliable stratification using blood samples from individuals gathered at a single time point [16].

DNA methylation is a widely studied epigenetic mechanism involving the addition of methyl groups to the DNA molecule, typically at cytosine-phosphate-guanine (CpG) dinucleotides. These modifications are involved in the regulation of gene expression and are influenced by both genetics and the environment [17]. Though DNA methylation is dynamic across the life-course, the short-term variability in adults is thought to be relatively stable, marking it as a potentially useful signature of exposure [18–20]. Through epigenome-wide association studies (EWAS), DNA methylation signals at individual CpG sites have been associated with various health and lifestyle factors, permitting the creation of methylation-based phenotypic predictors and signatures [21–24]. Recently, a large-scale EWAS of serum CRP in adults (*n* = 8863 and 4111 of European and African ancestries, respectively) identified DNA methylation correlates of chronic low-grade inflammation [25]. Using 7 CpG sites from this data, an inflammation-related epigenetic risk score was recently created and applied in a developmental framework investigating inflammation and child and adolescent mental health [26].

In a longitudinal study of older adults (the Lothian Birth Cohort 1936) and a large, cross-sectional cohort (Generation Scotland), we utilise this inflammation-related epigenetic score and characterise its relationship with serum CRP levels, a genetic CRP score, and physical health and lifestyle traits that have previously been associated with CRP. We examine the longitudinal dynamics of serum CRP and the epigenetic score, establishing the stability of each over time, and if there are multiple typical trajectories characterising latent subgroups of individuals sharing a common profile. Finally, we assess the comparative associations of each predictor with cognitive ability.

## Methods

### Lothian Birth Cohort 1936

The Lothian Birth Cohort 1936 (LBC1936) is a longitudinal study comprising individuals born in 1936, most of whom completed the Scottish Mental Survey 1947 aged around 11 years. Full details on the recruitment and assessment protocols of the study have been described previously [27, 28]. Briefly, 1091 participants were recruited to the study aged around 70 years. To date, up to three further waves of testing in older age have been completed at intervals of around 3 years at mean ages of 73, 76, and 79. At each wave, data has been collected on a wealth of health outcomes, lifestyle factors, cognition, and biological measures.

### Generation Scotland: the Scottish Family Health Study

Generation Scotland (GS) is a family-based genetic epidemiology cohort sampled from the general population across Scotland. The recruitment protocol and cohort characteristics are described in detail elsewhere [29, 30]. Initially, 7953 individuals aged between 18 and 65 years were recruited between 2006 and 2011 from General Practitioner registries. Family members of these subjects aged between 18 and 99 years were then approached to participate. The final cohort comprised around 24,000 subjects. Data were collected on various cognitive, psychiatric, and health measures, and DNA samples were collected for genotyping and methylation profiling.

### Phenotype preparation

#### C-reactive protein

##### LBC1936

Serum CRP was measured from venesected whole-blood samples. Levels were quantified using both a low-sensitivity assay (mg/L) performed using a dry-slide immuno-rate method on an OrthoFusion 5.1 F.S analyser (Ortho Clinical Diagnostics). This assay cannot distinguish values less than 3 mg/L, thus all readings of < 3 mg/L were assigned a value of 1.5 mg/L [31].

##### Generation Scotland

CRP was quantified at the University of Glasgow using a commercial high-sensitivity assay on an automated analyser (c311, Roche Diagnostics, UK). Manufacturer’s calibration and quality control were employed. CRP data was available for 419 individuals. These samples had been selected as father/offspring pairs in a telomere length study.

#### Cognitive ability

##### LBC1936

Fluid-type cognitive ability encompasses the capacity to perform basic information processing and extemporary thinking tasks, rather than those involving acquired knowledge or experience. This cognitive domain typically exhibits a decline with age. Scores on different tests of fluid-type cognitive ability are typically highly correlated, indicative of a latent general cognitive ability factor. Here, we derived a single general fluid-type cognitive ability score (*g*_*f*_) for each participant from the first un-rotated principal component of a principal components analysis on six of the Wechsler Adult Intelligence Scale-III tests. These tests assessed four different cognitive domains: letter-number sequencing and digit span backwards (working memory), digit-symbol coding and symbol search (processing speed), matrix reasoning (non-verbal reasoning), and block design (constructional ability) [32]. This component explained 53% of the variance across the 6 tests, with individual test loadings ranging from 0.66 to 0.78. Full details on the testing protocol for the cognitive tests in LBC1936 have been reported previously [27, 33].

##### Generation Scotland

Similarly to LBC1936, *g*_*f*_ was obtained for each participant from the first un-rotated principal component of a principal components analysis of three tests of cognitive ability: logical memory, verbal fluency (executive function), and digit-symbol coding (processing speed). This component explained 50% of the variance across the three tests with test loadings ranging from 0.63 to 0.78. Logical memory was assessed using the Wechsler Memory Scale-III [34]. Verbal fluency and digit symbol-coding were tested using the Wechsler Adult Intelligence Scale III [32]. Additional information regarding the cognitive variables in GS has been described previously [35, 36].

#### Physical and lifestyle variables

##### LBC1936

The variables previously associated with CRP levels included in the analysis in LBC1936 were as follows: body mass index (BMI: the ratio of weight in kilogram divided by height in square metres), self-reported smoking status (current smoker, ex-smoker, never smoker), alcohol consumption in a typical week (units), and social deprivation. Social deprivation was measured using the Scottish Index of Multiple Deprivation (SIMD). The SIMD ranks geographical areas in Scotland based on current income, employment, health, education, skills and training, geographic access to services, housing, and crime. The SIMD provides a standardised measure of relative deprivation throughout Scotland.

##### **Generation Scotland**

The variables in GS were all measured as described for LBC1936, the only exception being smoking status in which ex-smokers were divided into those who had quit within the previous 12 months of their blood sample date and those who had quit prior to that.

### Inflammation-related poly-epigenetic score

Details on the DNA methylation preparation for each cohort are presented in Additional file 1. An inflammation-related poly-epigenetic score (DNAm CRP) was derived for each participant as described by Barker et al. [26]. Briefly, methylation beta values were extracted for the 7 CpG sites shown to have the strongest evidence of a functional association with serum CRP levels. These values were multiplied by their respective regression weights (corresponding to change in DNA methylation beta values per 1 unit increase in natural log-transformed CRP) taken from the largest EWAS of CRP to-date and summed to generate a single DNAm CRP score for each participant (Additional file 2: Table S1 [25]). All of the regression weights from the EWAS were negative, resulting in a higher DNAm CRP score (i.e. closer to zero) corresponding to a prediction of increased CRP levels. Of the 7 CpG sites included in the original measure, one was not available on the EPIC array and was therefore was unavailable in the GS data (cg06126421). A DNAm CRP score inclusive of the remaining 6 CpG sites was utilised in analyses for GS.

It should be noted that LBC1936 contributed 296 high-sensitivity CRP samples (from wave 2) to the EWAS of CRP from which the DNAm SCRP score was derived [25]. This may mean results from this cohort are overfitted; however, the LBC1936 individuals represent a small subset of the discovery sample (*n* = 8863), and the probes were selected to be highly significant so it is unlikely this had a significant impact on results.

### Genetic score for CRP

Information on genotyping for each cohort is presented in Additional file 1. An additive weighted genetic score for CRP was constructed in both cohorts from the 18 single nucleotide polymorphisms (SNPs) that passed the genome-wide threshold (*p* < 5 × 10^−8^) in the largest available genome-wide association study (GWAS) of CRP to date [23]. Weighted dosages were calculated by multiplying the dose of each risk allele by the effect estimate from the GWAS (Additional file 2: Table S2). An imputation quality score of > 0.8 was applied to the SNPs.

### Statistical analysis

Pearson correlations were calculated between serum CRP, the DNAm CRP score (and its component CpGs), and the genetic CRP score in each cohort. Inter-wave correlations and intraclass correlation coefficients were estimated for the DNAm CRP score and serum CRP over the four waves of follow-up in LBC1936 to assess the stability of the measures over time.

Linear mixed models were used to investigate the change in the DNAm CRP score and serum CRP over the four waves in LBC1936. Sex was included as a fixed effect, age (years) as the timescale, and participant ID as a random effect on the intercept. Latent class mixed models were used to account for potential population heterogeneity in the trajectories and to determine the number of differing trajectories in both the serum and DNAm CRP data. Initially, a model with a single class was run. Additional classes were then added (up to 5) until the optimal number of classes was established using the Bayes Information Criterion (BIC) and the mean posterior probability of belonging to each class (mean probability > 0.65).

To investigate the validity of the DNAm and genetic CRP scores as markers of inflammation, linear regression models were run with phenotypes that have previously been associated with circulating CRP levels: BMI [37], smoking [38–40], alcohol [41], and deprivation [42, 43]. Age and sex have previously been identified as strong determinants of circulating CRP levels and thus were included as covariates in these models. Each phenotype was additionally included as a covariate unless fitted as the dependent variable of interest. Linear regression models were then used to obtain the cross-sectional associations between *g*_*f*_ and serum CRP, the DNAm CRP score, and the genetic CRP score. Models were adjusted for the aforementioned correlated phenotypes. Methylation set was included as an additional covariate in GS (see Additional file 1). In LBC1936, models were conducted at wave 1 (age ~ 70) of the study. To determine which CpG site in the DNAm CRP score was most relevant to the cognitive association, fully adjusted models with each of the CpGs that comprise the score as independent variables were computed. Correction for multiple testing was applied using the false discovery rate (FDR *p* < 0.05) [44].

Linear mixed effect models, with the baseline DNAm CRP score or serum CRP included as a fixed effect interaction with age, were used to test the prediction of subsequent change in cognitive ability. Participant ID was fitted as a random effect on the intercept.

Numeric variables were scaled to have a mean of zero and a variance of 1. Serum CRP and alcohol intake data were log-transformed (natural log) prior to analyses due to positive skews in their distribution.

Statistical analysis was performed in R version 3.5.0 [45]. Linear mixed-effect models and latent class mixed models were implemented using the ‘lmerTest’ and the ‘lcmm’ package, respectively [46, 47].

## Results

### Cohort information

Descriptive statistics of all the variables used in analyses are presented in Table 1. LBC1936 is an older cohort than GS (LBC1936 wave 1: mean = 69.5 years; GS: mean = 50.9 years), with a more even balance between sexes (LBC1936: 49% female; GS: 58% female). LBC1936 had a higher mean CRP (5.26 mg/L) compared to GS (2.65 mg/L). The mean genetic score for CRP was 2.16 in LBC1936 and 2.15 in GS.

**Table 1:** Cohort characteristics

	***n***	Mean	Median	Range	SD
**LBC1936 wave 1**	1091	–	–	–	–
Age (years)	–	69.53	69.54	67.61, 71.29	0.83
Sex (% female)	49.8	–	–	–	–
CRP (mg/L)	1054	5.26	3.0	1.5, 90	6.78
DNAm CRP	894	− 0.019	− 0.019	− 0.024, − 0.015	0.0013
Genetic score	1005	2.16	2.16	1.23, 2.83	0.25
*g*_*f*_	1070	0.096	0.12	− 3.43, 3.017	1.0091
BMI (kg/m^2^)	1083	27.78	27.39	16.023, 48.52	4.36
Alcohol (units/week)	1063	9.93	6	0, 70	12.12
Smoking status	894	−	−	−	−
Current	102	−	−	−	−
Ex	375	−	−	−	−
Never	417	−	−	−	−
SIMD	1083	5342	5342	4, 6505	1907
**Generation Scotland**	7028	–			–
Age (years)	–	50.89	52.75	18, 93.3	12.96
Sex (% female)	58.2	–	–	–	–
CRP (ng/mL)	419	2.65	1.21	0.15, 63.25	5.59
DNAm CRP	7028	− 0.012	− 0.013	− 0.016, − 0.0089	< 0.001
Genetic score	7028	2.15	2.17	1.14, 2.91	0.26
*g*_*f*_	6880	0.0063	0.042	− 4.15, 3.89	0.99
BMI (kg/m^2^)	6988	26.93	26.15	14.78, 51.29	5.089
Alcohol (units/week)	6414	10.49	8	0, 73	11.017
Smoking status	6854	–	–	–	–
Current	1135	–	–	–	–
Ex	2167	–	–	–	–
Never	3552	–	–	–	–
SIMD	6693	3953	4456	3, 6505	1857

*LBC1936* Lothian Birth Cohort 1936, *CRP* C-reactive protein, *DNAm* DNA methylation, *g*_*f*_ general cognitive ability score, *SIMD* Scottish Index of Multiple Deprivation, *BMI* body mass index

### Correlation between serum CRP, DNAm CRP score, and genetic score

In both cohorts, the Pearson correlations between the DNAm CRP score and serum log(CRP) were moderate (LBC1936: *r* = 0.34, 95% CI [0.28, 0.4]; GS: *r* = 0.28 [0.19, 0.36]), with the DNAm CRP score showing a stronger correlation with serum CRP than any one of its composite CpGs in both cohorts (Additional file 3. Figures S1 and S2). In LBC1936, the correlation between the genetic score for CRP and serum CRP was 0.21 [0.15, 0.27], and in GS, it was 0.29 [0.2, 0.38]. The correlation between the DNAm CRP score and the genetic score was low in LBC1936 (*r* = 0.07 [0.06, 0.14]) and not significantly different to zero in GS (*r* = − 0.01 [− 0.03, 0.016]).

### Trajectories of the DNAm CRP score and serum CRP

**Fig. 1:**
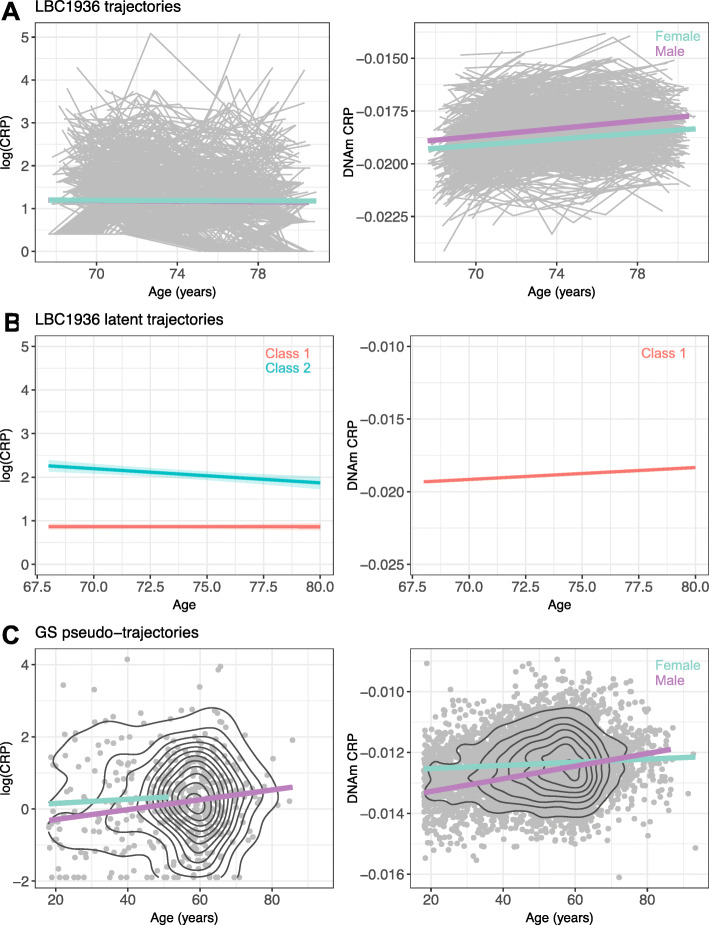
Trajectories of serum CRP and the DNAm CRP score over age. **a** Observed trajectories in the Lothian Birth Cohort 1936. Grey lines represent individual participant trajectories with regression lines shown for females and males in blue and purple, respectively. **b** Latent trajectories in Lothian Birth Cohort 1936. Latent classes are shown in red (class 1) and blue (class 2). **c** Pseudo-trajectories in Generation Scotland for CRP (*n* = 419) and the DNAm CRP score (*n* = 7028). Regression lines are as in (**a**). Black lines represent density. DNAm = DNA methylation; CRP = C-reactive protein

Plots of the observed trajectories of both serum log(CRP) and the DNAm CRP score in LBC1936 are shown in Fig. 1a. The observed trajectories of serum CRP in LBC1936 have been reported previously [48]. log(CRP) was found to decline over the 9 years of follow-up (*β* = − 0.014, SE = 0.005, *p* = 0.003). Conversely, the DNAm CRP score was found to increase by an average of 0.07 SD per year (SE = 0.004, *p* < 2 × 10^−16^). An interaction was identified between age and sex, indicating that the DNAm CRP score inclined faster over time in males compared to females (*β* = 0.018, SE = 0.007, *p* = 0.009). Plots of the pseudo-trajectories of serum log(CRP) and the DNAm CRP score in GS are shown in Fig. 1c. Both, log(CRP) and the DNAm CRP score increased with age (*β* = 0.011, SE = 0.004, *p* = 0.008; *β* = 0.013, SE = 0.0008, *p* < 2 × 10^−16^, respectively). Again, an interaction between age and sex was identified in the DNAm CRP score, with a more rapid rise in males than females (*β* = 0.017, SE = 0.002, *p* = 2 × 10^−16^).

To further investigate possible population heterogeneity in the trajectories, latent class analyses were conducted in LBC1936. The model with two latent classes had the best fit for serum log(CRP) (Additional file 2: Table S3). Though the BIC was minimised with four latent classes, this included a class of only 27 individuals and a low mean posterior class membership probability for class 1 (65.6%). The model with three classes also exhibited a low posterior probability of belonging to each latent class for class 2 (61.4%); therefore, the model with two classes was selected in which the mean probabilities for class membership were high—0.95 and 0.86 for class 1 and class 2, respectively. The two underlying mean trajectories are presented in Fig. 1b. In this model, class 1 (*n* = 811, 75.5%) exhibited a stable trajectory, with no evident change over the eighth decade (*β* = − 0.0025, SE = 0.005, *p* = 0.96). Class 2 (*n* = 263, 24.5%) showed a decline over time, likely indicating a subset of individuals exhibiting regression to the mean (*β* = − 0.032, SE = 0.008, *p* = 1 × 10^−4^). For the DNAm CRP score, class determination indicated the model with one class best fit the data. Here, a small elevation over time was identified (*β* = 0.06, SE = 0.005, *p* < 1 × 10^−5^).

### Stability of the DNAm CRP score and serum CRP

The intraclass correlation coefficient for serum CRP over the four waves of follow-up in LBC1936 was 0.72 (95% CI [0.69, 0.74], *p* < 2 × 10^−16^). For the DNAm CRP score, it was 0.82 ([0.75, 0.86], *p* < 2 × 10^−16^), ranging from 0.60 (cg27023597) to 0.94 (cg06126421) in the 7 CpG sites that comprised the score. The correlations of the DNAm CRP score and serum CRP between each of the four waves in LBC1936 are presented in Additional file 3: Figure S3. The inter-wave correlations of serum log(CRP) ranged from 0.3 (wave 1–wave 4) to 0.45 (wave 1–wave 2). The correlations of the DNAm CRP score between waves were stronger, ranging from 0.53 (wave 1–wave 4) to 0.75 (wave 2–wave 3), indicating an enhanced temporal stability.

### Associations with determinants of CRP

The associations between serum CRP, the DNAm CRP score, and the health and lifestyle phenotypes are presented in Table 2. No associations were found between the genetic score and any of the phenotypes in either cohort (Additional file 2: Table S4). Higher serum CRP was found to associate with a higher BMI and smoking status in both cohorts (LBC1936 BMI: *β* = 0.27, SE = 0.03, *p*_FDR_ = 2.1 × 10^−17^; smoking: log odds = 0.30, SE = 0.07, *p*_FDR_ = 1.4 × 10^−5^; GS BMI: *β* = 0.29, SE = 0.04, *p*_FDR_ = 3.3 × 10^−10^; smoking: log odds = 0.26, SE = 0.11, *p*_FDR_ = 0.03) but not with alcohol intake or social deprivation (*p* ≥ 0.14). In the subset of individuals with measured CRP in GS, the DNAm CRP score associated with BMI (*β* = 0.15, SE = 0.06, *p*_FDR_ = 0.019) and alcohol intake (*β* = 0.19, SE = 0.06, *p*_FDR_ = 0.003) but not with smoking (*p* = 0.29). In LBC1936 and the full GS cohort, a higher DNAm CRP score robustly associated with a higher BMI (LBC1936: *β* = 0.14, SE = 0.04, *p*_FDR_ = 2.4 × 10^−4^; GS: *β* = 0.19, SE = 0.01, *p*_FDR_ = 2.4 × 10^−38^), increased alcohol intake (LBC1936: *β* = 0.12, SE = 0.03, *p*_FDR_ = 0.001; GS: *β* = 0.15, SE = 0.01, 9.6 × 10^−24^), a more deprived socioeconomic status (LBC1936: *β* = − 0.12, SE = 0.03, *p*_FDR_ = 7.2 × 10^−4^; GS: *β* = − 0.07, SE = 0.01, *p*_FDR_ = 1.4 × 10^−6^), and being a smoker (LBC1936: log odds = 0.54, SE = 0.07, *p*_FDR_ = 1.5 × 10^−12^; GS: log odds = 0.24, SE = 0.03, *p*_FDR_ = 1.5 × 10^−18^).

**Table 2:** Associations between physical and lifestyle traits, and serum CRP and the DNAm CRP score

Lothian Birth Cohort 1936												
CRP					DNAm CRP							
	*β*	SE	*P*	FDR *P*	*β*		SE			*P*		FDR *P*
BMI	0.27	0.03	**3.1 × 10**^**−18**^	**2.1 × 10**^**−17**^	0.14		0.04			**8.1 × 10**^**−5**^		**2.4 × 10**^**−4**^
Alcohol	− 0.004	0.03	0.26	0.41	0.12		0.03			**5.9 × 10**^**−4**^		**0.001**
SIMD	− 0.05	0.03	0.14	0.25	− 0.12		0.03			**2.7 × 10**^**−4**^		**7.2 × 10**^**−4**^
Smoking	0.30	0.07	**4.2 × 10**^**−6**^	**1.4 × 10**^**−5**^	0.54		0.07			**2.7 × 10**^**−13**^		**1.5 × 10**^**−12**^
Generation Scotland												
CRP					DNAm—subset with measured CRP				DNAm—full dataset			
	*β*	SE	*P*	FDR *P*	*β*	SE	P	FDR P	*β*	SE	*P*	FDR *P*
BMI	0.29	0.04	**7.2 × 10**^**−11**^	**3.3 × 10**^**−10**^	0.15	0.06	**0.009**	**0.019**	0.19	0.01	**8.7 × 10**^**−40**^	**2.4 × 10**^**−38**^
Alcohol	0.01	0.07	0.83	0.91	0.19	0.06	**0.001**	**0.003**	0.15	0.01	**7.1 × 10**^**−25**^	**9.6 × 10**^**−24**^
SIMD	0.03	0.05	0.53	0.64	− 0.04	0.06	0.45	0.64	− 0.07	0.01	**3.7 × 10**^**−7**^	**1.4 × 10**^**−6**^
Smoking	0.26	0.11	**0.01**	**0.03**	0.16	0.11	0.17	0.29	0.24	0.03	**1.7 × x10**^**−19**^	**1.5 × 10**^**−18**^

Log odds are presented for smoking. Analysis was run at wave 1 of the Lothian Birth Cohort 1936 (*n* = 1091). Significant associations are highlighted in bold

*SIMD* Scottish Index of Multiple Deprivation, *BMI* body mass index, *CRP* C-reactive protein, *DNAm* DNA methylation, *SE* standard error, *FDR* false discovery rate

### Associations with cognitive ability

The associations between the individual predictors—serum CRP, the DNAm CRP score, and the genetic score—and *g*_*f*_ are presented in Table 3. CRP has previously been found to associate with cognitive ability cross-sectionally at wave 1 of LBC1936 [49]. In fully adjusted models in LBC1936, neither serum CRP nor the genetic CRP score was associated with *g*_*f*_ (*p*_FDR_ ≥ 0.16), but an inverse association was identified with the DNAm CRP score (*β* = − 0.08, SE = 0.03, *p*_FDR_ = 0.04). Similar results were found in GS, with no association with serum CRP or the genetic score (Table 3, *p*_FDR_ ≥ 0.71) but a higher DNAm CRP score associated with poorer cognitive ability (*β* = − 0.04, SE = 0.01, *p*_FDR_ = 0.04). The association between *g*_*f*_ and each of the CpG sites within the DNAm CRP score is presented in Additional file 2: Table S5. In both cohorts, cg18181703 (*SOCS3*) was associated with *g*_*f*_ with a larger effect size than that of the DNAm CRP score, suggesting this locus is particularly important in driving the observed cognitive associations.

**Table 3:** Associations between *g*_*f*_ and individual predictors

	Standardised *β*	SE	Raw *P*	FDR *P*
Lothian Birth Cohort 1936				
log(CRP)	− 0.033	0.03	0.26	0.41
Genetic score	0.052	0.03	0.08	0.16
DNAm CRP	− 0.084	0.03	**0.01**	**0.04**
Generation Scotland				
log(CRP)	− 0.016	0.05	0.74	0.77
Genetic score	0.007	0.01	0.57	0.71
DNAm CRP	− 0.035	0.01	**0.01**	**0.04**

Significant associations are highlighted in bold. Analysis was run at wave 1 of LBC1936 (*n* = 1091)

*CRP* C-reactive protein, *DNAm* DNA methylation, *SE* standard error, *FDR* false discovery rate

The longitudinal associations between baseline serum CRP and DNAm CRP score and *g*_*f*_ in LBC1936 are presented in Additional file 2: Table S6. There was no evidence to suggest either CRP or the DNAm CRP score at wave 1 of LBC1936 was predictive of subsequent change in *g*_*f*_ over the 4 years of follow-up (*p* ≥ 0.69).

## Discussion

We identified discrepant trajectories of serum CRP with age across two cohorts, whereas the DNAm CRP score was invariably found to increase with age and to do so more rapidly in males than in females. The DNAm CRP score additionally showed robust associations with health and lifestyle variables previously found to impact CRP levels. We found that a higher DNAm CRP score associated with lower cognitive function; however, neither baseline serum CRP nor the DNAm CRP score was associated with longitudinal change in cognitive ability.

Whereas ageing is considered to be linked to systemically raised inflammation [4], we identified divergent dynamics of serum CRP in the assessed cohorts, with both increasing and declining trajectories seen as a function of age. Contrastingly, we identified congruous increases in the DNAm CRP score in relation to age. In LBC1936, the increase per year was greater than that seen in GS, likely due to the inclusion of only older individuals within the cohort who may be more likely to experience a progressive elevation in inflammation [4, 50]. Furthermore, we consistently found a significant interaction between the DNAm CRP score and sex, with males having a steeper incline compared to females. This is conceivably due to men having a lower life-expectancy, and thus accelerated immune dysregulation, captured by the inflammation-related epigenetic score. Latent class analysis suggested the majority of individuals in LBC1936 exhibited stable dynamics of serum CRP over the eighth decade, with a slight decline identified in a smaller subgroup. The decline is likely due to a regression to the mean and highlights the acute nature and serum CRP and the challenges of utilising it as a biomarker of chronic systemic inflammation in studies where it is quantified only once, or even at multiple time-points with large sampling intervals across the life-course of a longitudinal study. Contrastingly, the analysis of the DNAm CRP score indicated a consistent population trajectory that increased over time. Moreover, the DNAm CRP score had higher inter-wave correlations and test-retest coefficients and more robust associations with the determinants of serum CRP, indicating it had less associated error than phenotypic CRP. As DNAm is considered relatively stable, the DNAm CRP score could conceivably be regarded as a cumulative, composite measure of inflammation akin to the HbA1c test typically utilised in diabetic patients to obtain a ~ 3-month average blood glucose recording [51]. The DNAm CRP score may then have the potential to provide a more sensitive biomarker of chronic inflammation, overcoming the noise that the phasic nature of serum CRP introduces, allowing for more reliable analyses of chronic inflammatory variance and its associative relationships.

In both cohorts, we reported an inverse association between cognitive ability and the DNAm CRP score, but no association with the genetic score or measured CRP. It seems this association is largely driven by cg18181703 (*SOCS3*). This site has previously been associated with higher scores on tests of vocabulary and speed of information processing in an EWAS meta-analysis of cognitive abilities (LBC1936 contributed to this study [52]), as well as with type 2 diabetes and BMI—both strong correlates of circulating CRP levels [53, 54]. *SOCS3* itself is involved in regulating pro-inflammatory cytokine signalling, and its expression has been found to be upregulated in the brains of patients with Alzheimer’s disease, suggesting it may be an important site in linking peripheral metabolic and inflammatory processes with central pathology and function [55]. Our results are consistent with a recent study identifying a negative association between the DNAm CRP score at birth and cognitive function in early life [26]. This, coupled with our results from GS, whose participant ages span early-adulthood to later-life, suggests inflammation and cognition may be related across the life-course rather than necessarily exclusively in older age. The larger effect size in LBC1936, however, suggests the association becomes more pronounced in older adults. Neither serum CRP nor the DNAm CRP score were found to associate with longitudinal change in cognitive ability over time indicating no predictive relationship between either measure of inflammation and cognitive decline. Previous studies have suggested that inflammation in middle age, rather than later life, may be a more powerful determinant of age-related cognitive change, and it would be interesting to utilise the DNAm CRP score in a longitudinal cohort spanning this age range to examine this hypothesis further [12, 56].

The strengths of this study include the large sample sizes and the range of longitudinal data available. We demonstrate that the DNAm CRP score could provide a proxy measure when CRP itself is not quantified, allowing for the investigation of inflammation in cohorts with only methylation data available. Limitations include the typically healthy nature of the two cohorts meaning our findings may not extrapolate to a more general population. Additionally, serum CRP is not directly produced by immune cells, and thus, it is, in itself, a proxy marker of inflammation. A chronic signature of inflammation might also manifest in whole blood through changes in cell type proportions which should also be considered in future analyses. While the DNAm CRP score may be capturing a more reliable picture of inflammation than measured CRP, it would be desirable to investigate its relationship with a panel of inflammatory mediators and to create epigenetic scores of other inflammatory biomarkers to test their comparative performance. Finally, no causal analysis was conducted in this study, so it remains to be determined (a) if CRP has a direct effect on methylation or indeed the opposite is true, though this has begun to be addressed elsewhere [25, 57]; and (b) if inflammation-related DNAm is causal of poorer cognition, vice-versa, or both are influenced by a confounding factor.

## Conclusion

Research into the complex relationship between systemic inflammation and cognitive ability relies upon accurate characterisation of inflammatory mediators to enable reliable conclusions. Here, we show that an inflammation-related poly-epigenetic score may provide a more stable index of chronic, low-grade inflammation in comparison with serum CRP itself. We found the epigenetic score associated more robustly with cognitive ability compared to the measured phenotype, demonstrating the potential value in using epigenetic information in place of labile phenotypes. DNAm signatures of acute inflammatory markers may provide a better signature of chronic inflammation, allowing for more reliable stratification of individuals, and thus clearer inference of its association with incident health outcomes.

## Supplementary information

**Additional file 1.** Supplementary methods.

**Additional file 2: Table S1.** CpG sites and relative weights (from Lighthart et al.) used to generate the DNAm CRP score. **Table S2.** SNPs and relative weights (from Dehghan et al.) used to generate the genetic score for CRP. **Table S3.** Summary of latent class analyses for log(CRP) and the DNAm CRP score in the Lothian Birth Cohort 1936. **Table S4.** Associations with determinants of CRP and the genetic CRP score. **Table S5.** Association between individual CpG sites and general cognitive ability (*g*_*f*_*)* in the Lothian Birth Cohort 1936 and Generation Scotland. **Table S6.** Longitudinal associations between the baseline DNAm CRP score and serum CRP and general cognitive ability (*g*_*f*_) over the four waves of follow-up in the Lothian Birth Cohort 1936.

**Additional file 3. Figure S1.** Pearson correlations between serum CRP, the DNAm CRP score and the genetic sco**re. Figure S2.** Correlations between individual CpGs comprising the DNAm CRP score and serum CRP in the Lothian Birth Cohort 1936 and Generation Scotland. **Figure S3.** Inter-wave Pearson correlations DNAm CRP score and serum CRP in Lothian Birth Cohort 1936.

## Data Availability

LBC1936 data are not publicly available due to them containing information that could compromise participant consent and confidentiality. LBC1936 data are available on request from the Lothian Birth Cohort Study, Centre for Cognitive Ageing and Cognitive Epidemiology, University of Edinburgh. According to the terms of consent for Generation Scotland participants access to data must be reviewed by the Generation Scotland Access Committee. Applications should be made to access@generationscotland.org.

## Abbreviations

CRP: C-reactive protein
CpG: Cytosine-phosphate-guanine
EWAS: Epigenome-wide association study
LBC1936: Lothian Birth Cohort 1936
GS: Generation Scotland
g_f_: General fluid-type cognitive ability
SIMD: Scottish Index of Multiple Deprivation
DNAm: DNA methylation
SNP: Single nucleotide polymorphism
GWAS: Genome-wide association study
FDR: False discovery rate
