# Supplementary material for: Characterisation of an inflammation-related epigenetic score and its association with cognitive ability
Source: Clin Epigenetics. 2020 Jul 27;12:113. doi: 10.1186/s13148-020-00903-8 (PMC7385981; doi:10.1186/s13148-020-00903-8)
Supplement: Supplementary file 1 — Additional file 1. Supplementary methods. [file 13148_2020_903_MOESM1_ESM.docx]

**Additional File 1**. Supplementary methods.

**DNA methylation preparation**

- *LBC1936*

Detailed information on the DNA methylation profiling of LBC1936 has been reported previously (1, 2). DNA methylation was measured at 485,512 CpG sites from whole-blood samples using the Illumina Human-Methylation450 BeadChip at the Edinburgh Clinical Research Facility. Quality control procedures were performed to remove low-quality samples (inadequate hybridisation, bisulfite conversion, nucleotide extension and staining signal) and probes with a low detection rate (<95% at p < 0.01) and low call rate (<450,000 probes detected at p<0.01). Samples where predicted, and reported, sex did not match, and probes on the sex chromosomes, were additionally excluded. Methylation data were available for 895 individuals at Wave 1 of the LBC1936 study.

- *Generation Scotland*

Genome-wide DNA methylation (DNAm) was profiled in samples derived from blood collected between 2006 and 2011 using the Illumina Human-MethylationEPIC BeadChip. The methylation arrays were run in two separate sets. The present study includes analysis on methylation data from 7,028 individuals in total (2,578 unrelated individuals in the first set and 4,450 unrelated individuals in the second set). Quality control steps for both sets have been fully reported previously (3). Briefly, ShinyMethyl was used to plot the log-median intensity of methylated versus un-methylated signal per array and outliers were excluded upon visual inspection (4). Samples in which 1% of CpGs had a detection p-value in excess of 0.05, probes with a bead count of <3 in more than 5 samples, probes in which 5% of samples had a detection p-value of >0.05, and those where predicted and recorded sex diverged were also removed.

**Genotyping**

- *LBC1936*

DNA samples were genotyped at the Edinburgh Clinical Research Facility using the Illumina 610 Quadv1 array. Preparation and quality control steps have been reported previously (5). Individuals were excluded on the basis of unresolved sex discrepancies, relatedness, and evidence of non-Caucasian descent. SNPs were excluded if they had a call rate ≤0.98, minor allele frequency ≤0.01, and Hardy-Weinberg equilibrium test with P ≤0.001.

- *Generation Scotland*

Genotyping was carried out using the Illumina HumanOmniExpressExome-8 v1.0 DNA Analysis BeadChip at the Wellcome Trust Clinical Research Facility (6). The arrays were imaged on an Illumina HiScan platform and genotypes were called automatically using GenomeStudio Analysis software v2011.1. SNPs with a minor allele frequency ≤0.01 and Hardy-Weinberg equilibrium test with P < 1x10^-6^ were excluded.

**References**

1. Marioni RE, Shah S, McRae AF, Chen BH, Colicino E, Harris SE, et al. DNA methylation age of blood predicts all-cause mortality in later life. Genome biology. 2015;16:25.

2. Shah S, McRae AF, Marioni RE, Harris SE, Gibson J, Henders AK, et al. Genetic and environmental exposures constrain epigenetic drift over the human life course. Genome research. 2014;24(11):1725-33.

3. Madden RA, McCartney DL, Walker RM, Hillary RF, Bermingham ML, Rawlik K, et al. Birth weight predicts psychiatric and physical health, cognitive function, and DNA methylation differences in an adult population. bioRxiv. 2019:664045.

4. Fortin J-P, Fertig E, Hansen K. shinyMethyl: interactive quality control of Illumina 450k DNA methylation arrays in R. F1000Res. 2014;3:175-.

5. Davies G, Tenesa A, Payton A, Yang J, Harris SE, Liewald D, et al. Genome-wide association studies establish that human intelligence is highly heritable and polygenic. Molecular psychiatry. 2011;16(10):996-1005.

6. Gunderson KL. Whole-genome genotyping on bead arrays. Methods in molecular biology (Clifton, NJ). 2009;529:197-213.
