# Supplementary material for: Characterisation of an inflammation-related epigenetic score and its association with cognitive ability
Source: Clin Epigenetics. 2020 Jul 27;12:113. doi: 10.1186/s13148-020-00903-8 (PMC7385981; doi:10.1186/s13148-020-00903-8)
Supplement: Supplementary file 3 — Additional file 3. Figure S1. Pearson correlations between serum CRP, the DNAm CRP score and the genetic score. Figure S2. Correlations between individual CpGs comprising the DNAm CRP score and serum CRP in the Lothian Birth Cohort 1936 and Generation Scotland. Figure S3. Inter-wave Pearson correlations DNAm CRP score and serum CRP in Lothian Birth Cohort 1936. [file 13148_2020_903_MOESM3_ESM.docx]

**SUPPLEMENTARY FIGURES**

**Supplementary Figure 1.** Pearson correlations between serum CRP, the DNAm CRP score and the genetic score in the Lothian Birth Cohort 1936 and Generation Scotland. 95% confidence intervals are presented in square brackets.

LBC1936=Lothian Birth Cohort 1936; GS=Generation Scotland; CRP=C-reactive protein; DNAm=DNA methylation.


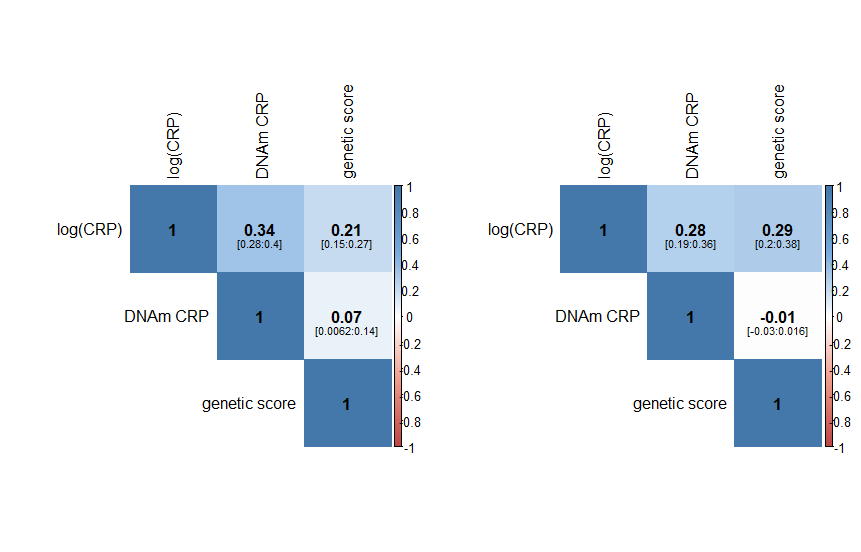


**GS**

**LBC1936**

**Supplementary Figure 2**. Plots of the correlations between individual CpGs comprising the DNAm CRP score and serum CRP in the Lothian Birth Cohort 1936 and Generation Scotland. Pearson correlations are presented in the upper diagonal and Spearman correlations are presented in the lower diagonal.


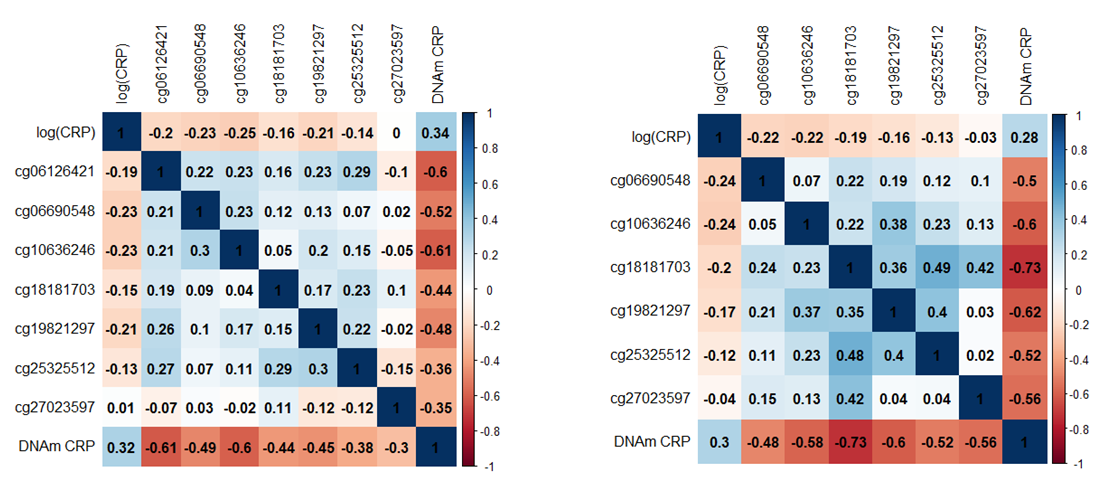


**GS**

LBC1936=Lothian Birth Cohort 1936; GS=Generation Scotland; CRP=C-reactive protein; DNAm=DNA methylation.

**LBC1936**

**GS**

**Supplementary Figure 3.** Inter-wave Pearson correlations DNAm CRP score and serum CRP in the Lothian Birth Cohort 1936. 95% confidence intervals are presented in square brackets.

CRP=C-reactive protein; W=wave; DNAm=DNA methylation
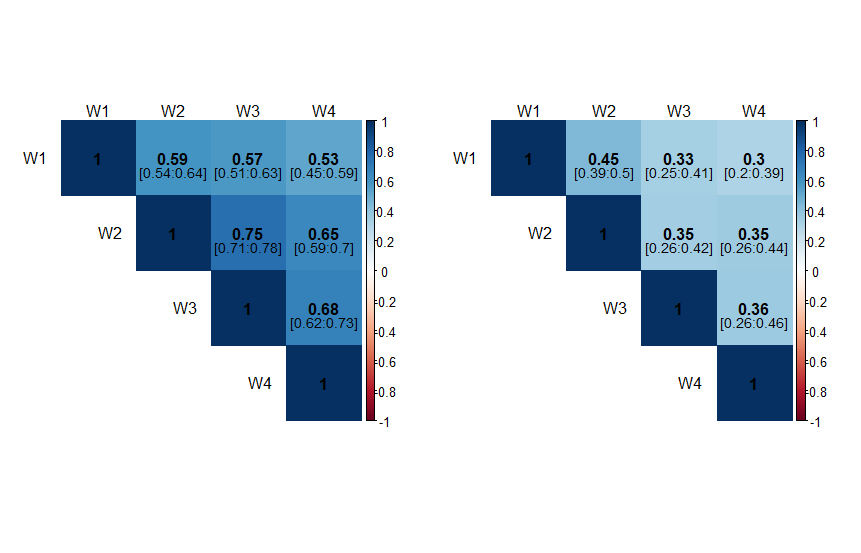


**DNAm CRP**

**Serum CRP**
